# Intensified upstream processing by a phosphate-regulated, auto-inducible expression system in *E. coli* W3110 for recombinant Fab production

**DOI:** 10.1186/s12866-026-04887-y

**Published:** 2026-03-11

**Authors:** Rüdiger Lück, Christian Zimmermann, Oliver Spadiut, Julian Kopp

**Affiliations:** Institute of Chemical, Environmental and Bioscience Engineering, TU Wien, Getreidemarkt 9, 1060 Vienna, Austria

**Keywords:** Intensified biomanufacturing, *Escherichia coli*, phoA expression system, Recombinant protein production, Fragment antigen binding

## Abstract

**Background:**

*Escherichia coli* is a microbial expression system widely spread in industrial manufacturing systems for the production of biotherapeutics. Most of these recombinant protein production processes are established in fed-batch operation mode. While many sectors of the chemical industry have implemented intensification strategies, the bio-pharmaceutical sector lacks a profound change towards intensified operations due to a decrease of cellular productivity during extended cultivation periods.

**Results:**

In this study, intensified bioreactor cultivations of an auto-inducible *E. coli* strain W3110 as a promising candidate for the production of a recombinant fragment antigen binding (Fab) are demonstrated. The main goal was to investigate the impact of the natural phosphate limitation on the activity of the alkaline phosphatase promoter (phoA) in terms of cell-specific productivity in different processing modes. Following a cell-physiological characterization in fed-batch operation, the transition to intensified upstream processing modes, such as repetitive fed-batch and chemostat was established successfully. A comprehensive understanding of phosphate limitation and precise control of critical parameters, such as growth rate and phosphate-to-substrate uptake enabled the intensified process to match specific product titers and surpass process efficiency (in terms of space-time yield) compared to fed-batch mode.

**Conclusions:**

The auto-inducible phoA promoter in *E. coli* W3110 enabled stable Fab production under intensified bioprocess operations. These findings highlight the suitability of this expression system for repetitive fed-batch and chemostat operations to support broader adoption of intensified strategies in the bio-pharmaceutical industry.

**Supplementary Information:**

The online version contains supplementary material available at 10.1186/s12866-026-04887-y.

## Background

Antibody-based therapeutics have become a driver of drug research over the last decade [1]. While only two antibodies were approved by the U.S. Food and Drug Administration (FDA) in the year 2013, there were twelve newly approved antibodies in 2023, 13 in 2024 and ten more in 2025 [2–4]. Fragment antigen binding (Fab) is a type of antibody that is composed of only the variable region of a full-length antibody. While keeping the functional properties of a full-length antibody, a Fab lacks the Fc domain leading to a much smaller molecular weight (Fab $\thicksim 50$ kDa, mAb $\thicksim 150$ kDa), higher tissue penetration and reduced immunogenic effects for patients [5]. The microbial host *Escherichia coli* (*E. coli*) is well established for the periplasmic production of Fabs as the oxidizing conditions in the periplasmic space allow for the required formation of disulfide bonds [6–8]. Furthermore, *E. coli* offers several benefits over mammalian hosts for Fab production. The fast growth on minimal media, ease of genetic manipulation, possibility of high-cell density cultivations, high product yields and an overall cost-effective production are some advantages of this host [9]. Common *E. coli* strains used for producing Fab fragments are B and K-12 strains, such as BL21(DE3), HMS174(DE3) and W3110 [10–12]. These strains are favored due to their ability to achieve high cell density cultivations, robust cell growth and suitability of periplasmic expression via a signal peptide [13]. The strain BL21(DE3) in combination with the T7 promoter is the expression system of choice for recombinant antibody production, as it’s tightly controlled by the T7 RNA polymerase, protease deficient and yielding in high levels of target gene expression [14]. The K-12 strain W3110 is characterized by moderate expression levels and high genetic stability making it an industrially relevant strain [15, 16]. Although W3110 does not have protease deficiency (Lon protease) like B strains, it is less sensitive to toxic metabolic by-products, environmental stress and maintains growth at low oxygen levels making it favorable for high cell density cultivations at scale [17]. Established promoters for regulating Fab expression are Isopropyl $\beta$-D-1-thiogalactopyranoside (IPTG) inducible promoters (e.g., T7lac), arabinose inducible promoters (e.g., araBAD), temperature regulated promoters (e.g., pL) and auto-inducible promoters (e.g., phoA) [8, 18]. Despite the advantages of the widely applied T7lac system for Fab production, its strong induction mechanism can overload the host expression system and secretory machinery leading to metabolic stress, formation of protein aggregates and increased genetic escape rates impairing recombinant protein synthesis [19, 20]. Consequently, long-term stability of *E. coli* cultivations remains as a major issue for strong inductive production systems [21].

A recent publication highlighted, that the application of auto-inducible promoters improves soluble Fab expression in the periplasm [6]. The auto-inducible phoA promoter responds to the natural phosphate starvation of the cells [22]. Phosphate (PO_4_) is an essential element of the cellular energy metabolism, nucleic acid synthesis and membrane transport mechanisms and signal transduction [23, 24]. *E. coli* regulates the intracellular PO_4_ level through the Pho regulon, whereby the PhoR-PhoB two-component system acts as a global regulatory circuit. Under PO_4_ excess conditions, diffusion-based passive transport into the cell is sufficient and the Pho regulon remains inactive. PO_4_ starvation ($<4\mu M$) activates the transport mechanisms [25]. In direct comparison with the commonly used T7lac promoter system, the absence of a strong inducer lowers the metabolic burden on the cellular expression machinery [26]. Thereby, the formation of misfolded aggregated proteins, so called inclusion bodies, is reduced, while favoring soluble expression with the phoA system [27, 28]. Another advantage of the auto-inducible promoter is, that the system does not require intervention (adding the inducer) in the process, lowers the risk of contamination and material costs are saved [26]. Previous studies also showed that a lower feeding rate increases the periplasmic Fab yield, since leakage through the outer cell membrane is reduced [15, 29]. Soluble Fab expression minimizes downstream processing efforts since no solubilization and refolding of the aggregated protein is required.

Even though process intensification strategies were implemented in various sectors since the 1970s, biopharmaceutical production is mainly implemented in batch/fed-batch mode [30–32]. Hereby, process intensification offers several advantages: reduced equipment downtimes, less bulk chemical usage, higher space-time yields and shrinking of equipment sizes [33]. Repetitive fed-batch (RFB) and chemostat are often applied as intensified operation strategies [34, 35], though chemostat operation can be unsuitable for product-inhibited systems [36]. Therefore, cascaded processing strategies were developed to circumvent malfunctioning protein expression in a single chemostat bioreactor occurring either due to cell-toxic inducers or cell-toxic protein expression [37, 38]. A cascaded processing strategy spatially separates biomass and product formation in two bioreactors, at the cost of increased operational complexity. The RFB strategy, on the other hand, offers a compromise between fully continuous biomanufacturing and the traditional fed-batch mode. Hereby, a sequence of fed-batch cultivations is executed reducing cleaning and sterilization cycles (technical details provided in Bioreactor cultivations section) [39]. Despite the advantages of intensified bioprocessing, industrially relevant intensified Fab production processes remain scarce, and to our knowledge, only a few intensified laboratory-scale cultivations have been reported [19, 40]. We believe this is due to the fact that mainly inducible systems have been historically used for Fab production. The metabolic burden of recombinant protein production leads to unstable equilibria caused by metabolic shifts, subpopulation formation due to high genetic escape rates and a loss of cellular productivity during intensified bio-processing [41]. Interestingly, Schuhmacher et al. reported the possibilities of PO_4_ limited fed-batch processing to control cell growth and oxygen uptake [42].

However, auto-inducible expression systems remain underexplored for intensified Fab production with no established strategy for stable PO_4_ limited operation. In particular, it remains unclear whether PO_4_-dependent auto-induction can be reliably controlled over extended cultivation times without compromising cellular physiology or productivity. In this study, an *E. coli* strain W3310 with a PO_4_-sensitive phoA promoter for the expression of a recombinant Fab was cultivated in various processing modes. We hypothesized that precise control of extracellular PO_4_ at a defined functional threshold is sufficient to reproducibly activate the phoA promoter while maintaining stable cellular physiology and cell-specific productivity across different processing modes. To test this hypothesis, the goals of this study were (1) to characterize the cellular physiology (specific growth rate, specific PO_4_ uptake rate) and the Fab productivity (specific titer, specific product formation rate) under PO_4_ limitation and (2) to apply this knowledge onto various intensified processing strategies using auto-induction by varying the harvest strategy and the feed composition. In general, the identification of quantitative physiological parameters and thresholds, such as PO_4_-dependent induction points, the stoichiometry of nutrient uptake mechanisms, growth–productivity dependencies and cellular adaptation limits, provides a mechanistic basis for translating cell physiology into process control variables.

## Methods

### Recombinant product

The target molecule was the antigen binding fragment (Fab) Ranibizumab consisting of a light and a heavy chain with similar molecular weight (23 kDa light chain, 25 kDa heavy chain) [43]. The light and heavy chain were linked with a single intermolecular disulfide bond. The correct assembly of heavy and light chain is a critical step, since the Fab is only biochemically active after the right constitution as a functional heterodimer and the presence of a tridimensional antigen-binding site [6, 12].

### Strain

The *Escherichia coli* strain W3110 (DSMZ, Braunschweig, Germany) with a genetically engineered pAT153 plasmid vector was used for all cultivations. After co-expression of the Fab genes in the cytoplasm, translocation of the antibody chains to the periplasm was triggered by the enterotoxin II (STII) signal sequence. The oxidizing conditions of the periplasmic space allowed correct formation of the disulfide bonds. Fab genes were encoded in the pAT vector, where the gene coding the $$\beta $$-lactamase ($$Amp^R)$$ was exchanged with the insert of interest. The alkaline phosphatase (phoA) promoter controls the transcription of the Fab genes [28]. The *E. coli* phoA strain is an auto-induced expression system, where the promoter is regulated based on the extracellular phosphate level. Previous studies demonstrated the benefits of the phoA expression system for the production of Fabs or single-chain fragments variable (scFv) [6, 22].

### Bioreactor cultivations

The cultivations were carried out in laboratory bench-top bioreactor systems, whereby two systems were used: the Minifors 2 bioreactor system (max. working volume 2.5 L) and the Multifors 2 system (max. working volume 1 L; both Infors HT, Bottmingen, Switzerland). A defined minimal medium according to DeLisa [44] with an adapted concentration of inorganic phosphate (PO_4_) was used. The minimal media was supplemented with glucose as the main carbon source to an initial concentration of 20 g L^-1^. Tetracycline hydrochloride was chosen as a selection marker to prevent plasmid loss (10 mg/L). Cells were grown overnight in a Infors HR Multitronshaker (Infors, Bottmingen, Switzerland) using Ultra-Yield-Flasks (Thomson, Carlsbad, USA) with a fill volume of 0.5 L at 37°C, 230 rpm shaking frequency, 15 hours cultivation time. The pre-culture cell broth was concentrated 6-fold by centrifugation to achieve a starting biomass concentration of 1 g/L (5,000 rpm, 5 min, 4°C). The reactor was inoculated with this pre-culture cell broth, where 10% of the initial reaction volume was added as inoculum. Cells were growing during the batch phase at 35°C until all sugar in the initial media was depleted. Afterwards, an exponential feed (400 g/L glucose; Eq. 1) was started for further cell expansion and the reactor temperature was lowered to 30°C reducing cell stress [28]. Fed-batch (FB) experiments were conducted at various exponential, cell-specific feeding rates. The FB cultivation was harvested, when all extracellular phosphate was depleted, indicated by a drop of the carbon dioxide off-gas signal. Repetitive fed-batch (RFB) experiments were partially harvested at an extracellular PO_4_ concentration of 1 mM, while fresh media (300 mL) was added to the remaining cell broth at a 1:1 ratio. The fresh media contained no C-source, but inorganic salts, macro nutrients, trace elements and the selection marker as described before [44]. Chemostat experiments included a FB phase as well, until a biomass target concentration of 20 g/L dry cell weight (DCW) was reached. Subsequently, the continuous process phase was initialized by starting the bleed flow steadily removing cell broth from the reactor via a dip tube and supplying a tailored continuous feed to the reactor. A cascaded processing strategy was implemented using a two-stage bioreactor setup. The first bioreactor stage was operated in chemostat mode (as described above) and served primarily for biomass generation. The bleed from the first stage was continuously transferred to the second stage, which was supplied with an additional feed allowing the adjustment of the PO_4_/S ratio between 0-0.16 mM/mM. The aeration of the reactor vessel was done with pressurized air and oxygen on demand (2 vvm) through an orifice sparger at the reactor bottom. The stirrer speed was kept at 1,400 rpm for batch and FB phase, throughout the continuous phase the stirrer speed was adjusted to 1,100 rpm to maintain the adjusted volume. The process values of scales (base, feed), dissolved oxygen ($pO_2$), pH, temperature, aeration and off-gas concentrations were recorded in real-time through the process information managament systems eve (Infors HT, Bottmingen, Switzerland) and Lucullus PIMS (Securecell, Ursdorf, Switzerland). The level of $pO_2$ was tracked with an optical $pO_2$ probe (Visiferm DO225, Hamilton, Reno, NV, USA) controlled towards a lower limit of 40%, if needed pure oxygen was added to the air flow. Off-gas analytics were recorded either through a Bluesens and Bluevary sensor (BlueSens Gas analytics, Herten, Germany) measuring oxygen and carbon dioxide concentrations in the off-gas stream. The pH was monitored via an EasyFerm Plus pH electrode (Hamilton, Reno, NV, United States) and controlled at 7.2 via addition of 12.5% ammonia. All feed bottles were placed on scales for precise tracking of the feed rates. Samples were taken at the beginning of the process, at the end of batch phase, multiple times during the FB phase, and twice daily during the continuous phase.

### Analytical methods

Samples taken from the reactor were immediately cooled at 4°C and further processed. The biomass was quantified via determination of the optical density of the cell broth at 600 nm using a photometer (Genisys20, Thermo Scientific Waltham, MA, USA). The biomass concentration was also determined via DCW in triplicates gravimetrically. A volume of 1 mL cell broth was centrifuged (14,000 rpm, 10 min, 4°C) in pre-weighted micro reaction tubes, followed by washing of the cell pellet with 0.9% NaCl solution and a further centrifugation step. The washed cell pellets were dried in the oven at 120°C for at least 72 h. The supernatant of the first centrifugation step was collected for further analysis. Residual sugar, PO_4_ and metabolites concentrations in the supernatant were monitored at-line via colorimetric assay kits of the Cedex Bio HT Analyzer (Roche, Basel, Switzerland). The intracellular product quantification comprised several steps. First, the cell broth was centrifuged in 50 mL tubes (Greiner Bio-One, Kremsmünster, Austria) at 14,000 rpm for 20 min at 4°C. The cell pellet was resupended in 40 mL lysis buffer (20 mM NaH_2_PO_4_, 100 mM NaCl, pH 7.2) and cells were disrupted through high-pressure homogenization at 1200 bar for 4 passages (PandaPLUS, Gea AG, Germany). The crude cell lysate was centrifuged with the same settings and the supernatant containing the soluble product fraction was collected. Prior to the affinity HPLC analysis, the homogenized supernatant was pre-treated with a delipidation step preventing column fouling and then filtered (0.2 $\upmu$m) [45]. The HPLC analysis was done on a Vanquish Flex HPLC system (Thermo Fisher Scientific, USA) equipped with an affinity Protein L column (POROS CaptureSelect KAPPAXL 2.1mm x 30 mm, ThermoFisher, Carlsbad, CA, USA) and a pre-column filter (Waters ACQUITY UPLC Col. In-Line Filter Kit, Waters Corporation, Milford, MA, USA). The product was quantified by measuring purified standard of Ranibizumab in the range of 50-361 mg/L (TargetMol, Linz, Austria). For RT-qPCR analyses, the RNA was extracted from approximately 0.1 g of *E. coli* biomass. Cells were resuspended in RNAzol RT (Sigma-Aldrich) and disrupted using a FastPrep-24 homogenizer (MP Biomedicals, Santa Ana, CA, USA) with 1 mm glass beads in two cycles of 30 s at 6 m/s. After a 5 min incubation at room temperature, cell debris was removed by centrifugation (12,000 g, 5 min). The lysate was combined with ethanol and RNA was purified using the Direct-zol RNA Miniprep Kit (Zymo Research, Irvine, CA, USA), including DNase treatment to remove genomic DNA. RNA concentration and purity were assessed using a NanoDrop ONE (Thermo Fisher Scientific, Waltham, MA, USA). Reverse transcription was performed using 500 ng of total RNA and LunaScript RT SuperMix (NEB). The resulting cDNA was diluted 1:50 and quantified by RT-qPCR using Luna Universal qPCR Master Mix (NEB) with gene-specific primers. The relative transcript ratios were calculated using the Pfaffl method and *cysG* and *idnT* as reference genes [46].

## Results

State-of-the-art FB experiments were done in order to identify strain physiological parameters (e.g., cell-specific PO_4_ uptake, growth rate) and evaluate the cell-specific productivity. The FB experiments were further utilized as a benchmark against intensified processing strategies being RFB and chemostat.

### Strain physiological characterization in fed-batch operation

**Fig. 1:**
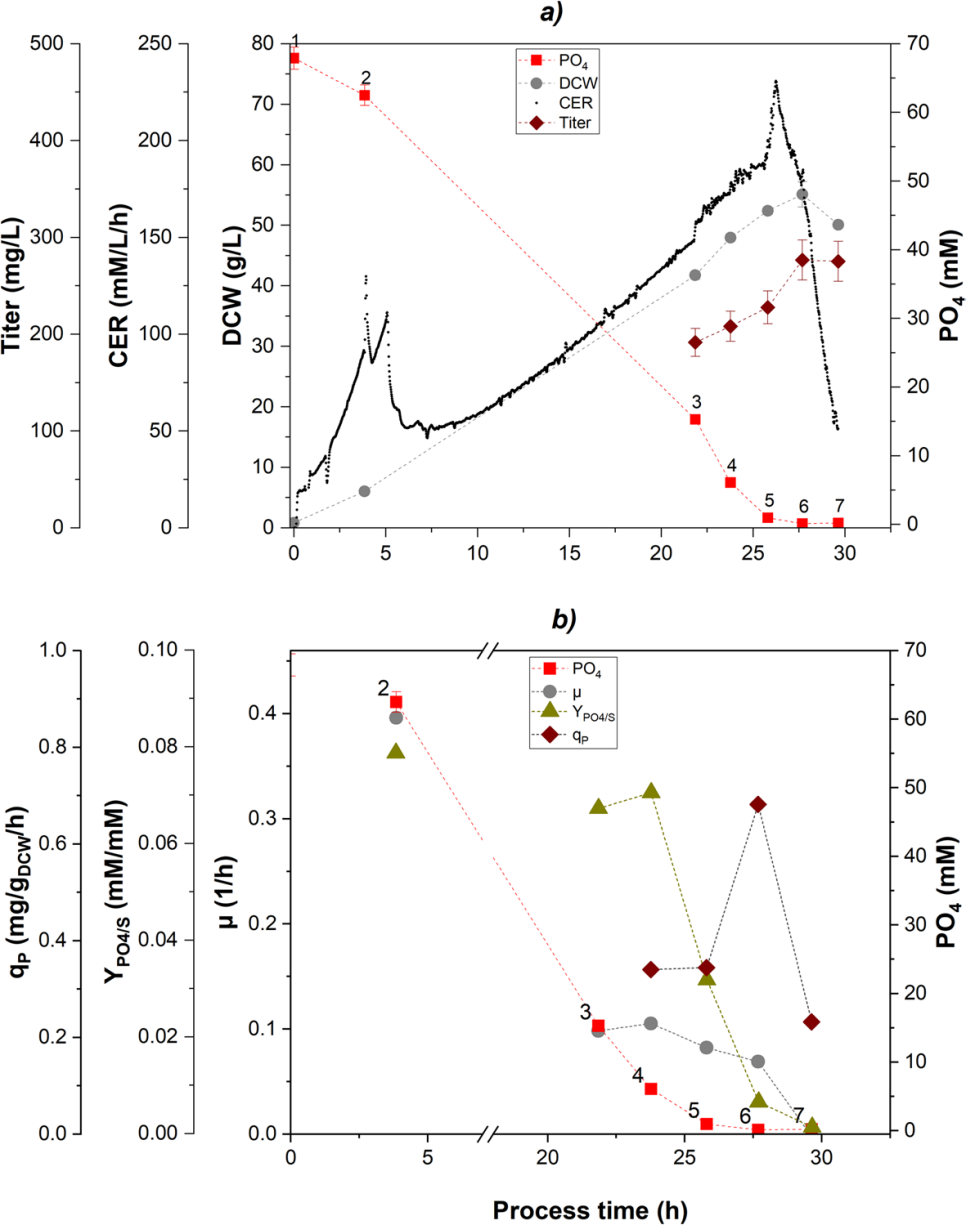
Fed-batch cultivation (FB 1): **a** after initial batch phase (sample 2) glucose was depleted and fed exponentially with a specific growth rate of $\mu =0.1 \; h^{-1}$ leading to exponential biomass growth (gray circles), cultivation terminated after full PO_4_ (red squares) limitation (sample 7) indicated by drop of CER signal (black dots), volumetric titer determined via HPLC (brown diamonds); **b** implications of extracellular PO_4_ level (red squares) on cell-specific growth rate (gray circles), PO_4_/substrate uptake (green triangles) and specific product formation rate (brown diamonds)

The starting point for process intensification was to investigate the impact of PO_4_ limitation on the cell-specific growth rate, PO_4_/substrate uptake, carbon recovery and the cell-specific productivity in FB operation. Three fed-batches FB 1, FB 2 and FB 3 with varied cell-specific feeding rates (corresponding growth rate $\mu _{set}$) were designed to determine PO_4_ uptake rates and product formation rates. Hereafter, the results of FB 1 are examined in detail. The biomass grew under C-limiting conditions up to a maximum of 55 g/L (Fig. 1a, sample 6). CO_2_ off-gas measurements and the obtained carbon dioxide evolution rate (CER) were utilized for monitoring the exponential cell growth. Followed by the exponential growth incline during PO_4_ excess, an elevated CER peak was recognized once extracellular PO_4_ limitation was reached (samples 5-6). The cultivation was intentionally prolonged beyond the decline of the CER to capture the full PO_4_ limitation response. The PO_4_/substrate uptake was constant ($Y_{PO4/S} = 0.07 \; mM/mM$) under PO_4_ excess (Fig. 1b). When the cultivation approached PO_4_ limitation between sample 4 (6.1 mM PO_4_) and sample 5 (1.0 mM PO_4_), the cellular PO_4_ uptake decreased indicated by a drop of the uptake ratio $Y_{PO4/S}$. Under carbon-limiting conditions during FB phase, the cell-specific growth rate $\mu _{set}$ was set constant to $0.1 \; 1/h$ resulting in an exponential biomass growth profile. However, the observed $\mu$ decreased under PO_4_ limitation and finally dropped to zero (sample 7). Following a decreased cell growth, glucose accumulated in the cell broth at the end of the cultivation (samples 6-7, Fig. S2). The carbon-recovery under PO_4_ excess was calculated by including the biomass/substrate yield $Y_{X/S}$ (0.50 C-mol/C-mol), the carbon dioxide/substrate yield $Y_{CO_2/S}$ (0.46 C-mol/C-mol) and the acetate/substrate yield $Y_{Ac/S}$ (0.04 C-mol/C-mol) (Fig. S1). The obtained carbon-recovery was determined between $0.9-1$ for non $PO_4$-limiting conditions. However, the carbon-recovery decreased below 0.9 when $PO_4$ limitation approached. $Y_{X/S}$ dropped since the cell growth ceased (0.02 C-mol/C-mol), while $Y_{CO_2/S}$ grew significantly (0.58 C-mol/C-mol).

**Fig. 2:**
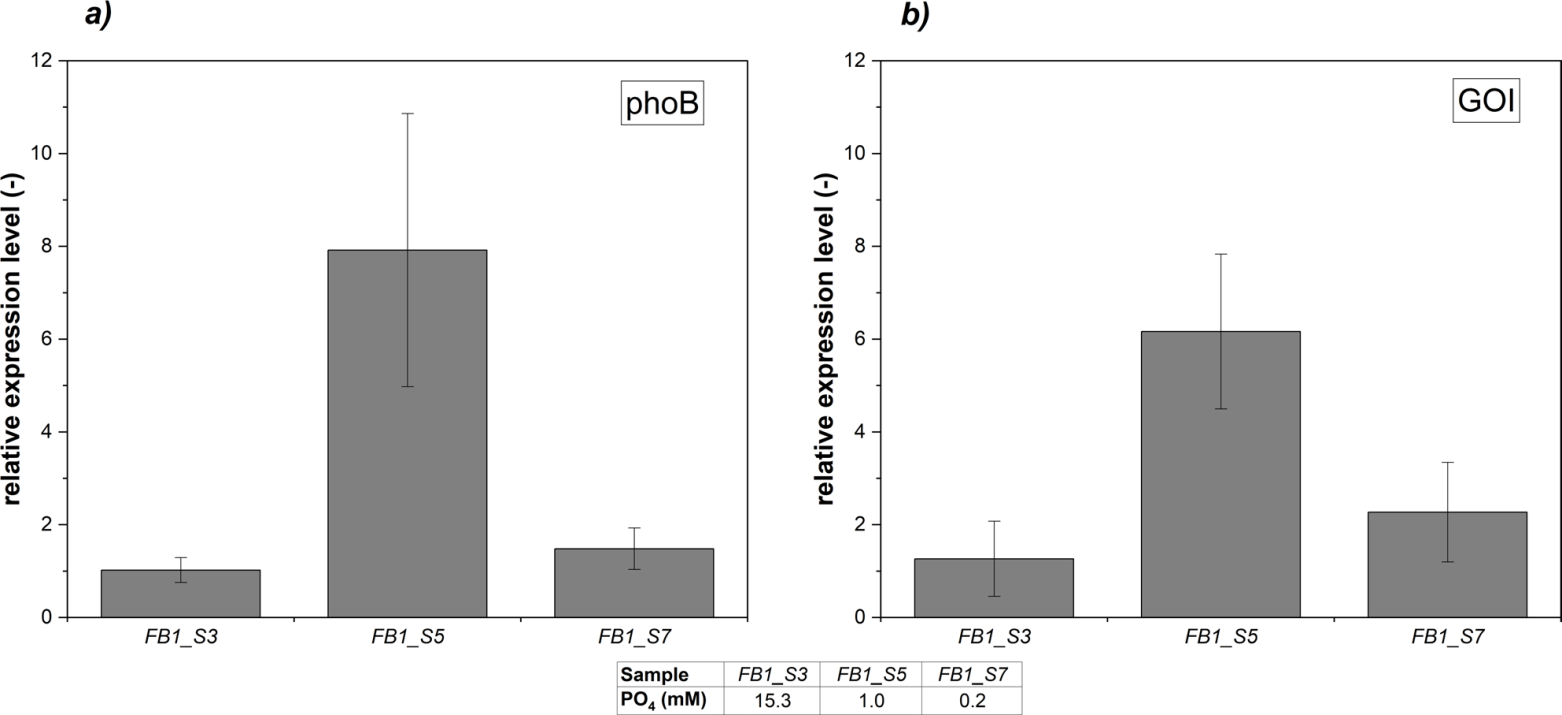
Relative expression levels of phoB gene and gene of interest (Fab) shown for selected FB1 samples, reference genes *idnT* and *cysG* used for normalization of expression levels, **a** relative expression levels of *phoB* acting as transcriptional response regulator for intracellular PO_4_ homeostasis, **b** relative expression levels of gene of interest (Fab)

The PO_4_ and $\mu$ dependency of Fab production was assessed for three different FB cultivations. The phoA promoter showed leaky Fab expression in all FB cultivations at levels of PO_4_ excess. The specific product formation rate $q_P$ was constant at $0.3\; mg/g_{DCW}/h$ and increased to $0.64\; mg/g_{DCW}/h$, when full PO_4_ limitation was approached (Fig. 1b). Varying the specific growth rate $\mu _{set}$ across the three cultivations resulted in different Fab productivities (Table 1). The highest specific titer $Y_{P/X}$ of $7.64 \; mg/g_{DCW}$ and a space-time yield (STY) of $6.59\; mg/L/h$ were obtained at $\mu _{set}=0.075\;1/h$ (FB 2). Although, FB 1 yielded in the lowest $Y_{P/X}$, the STY of $6.46\; mg/L/h$ was similar to FB 2 due to a shorter cultivation time. The average specific product formation rate $q_{P,\,avg}$ increased with a higher $\mu _{set}$. Across all FB cultivations, the onset of PO_4_ limitation was identified between 3.4 and 6.1 mM, as indicated by a decline of $Y_{PO4/S}$ (Fig. S3). Still, highest product formation rates of $q_{P,\,max}=\left[ 0.6-0.7\right] \;mg/g_{DCW}/h$ were observed only below 1 mM PO_4_ (defined here as a full PO_4_ limitation). RT-qPCR analyses of the *phoB* gene and the gene of interest (Fab) were performed to validate the relationship between extracellular PO_4_ availability and the cell-specific product formation rate $q_P$ observed in the FB cultivations. The relative expression levels of *phoB* encoding the response regulator of the Pho regulon mirrored the expression pattern of the Fab gene (Fig. 2). Under extracellular PO_4_ excess conditions, both *phoB* and Fab transcript levels remained low (FB1_S3). In contrast, full PO_4_ limitation resulted in a significant upregulation of both genes (FB1_S5). Upon prolonged exposure to full PO_4_ limitation the transcript levels declined (FB1_S7).

**Table 1:** Fed-batch cultivations with varied $\mu _{set}$ leading to different Fab productivities

Cultivation	$\boldsymbol{\mu }_{\boldsymbol{set}}$ (1/*h*)	Cultivation time (*h*)	Y_P/X_ ($\boldsymbol{mg/g}_{\boldsymbol{DCW}}$)	q_P,avg_ ($\boldsymbol{mg/g}_{\boldsymbol{DCW}}\boldsymbol{/h}$)	STY (*mg*/*L*/*h*)
FB 1	0.10	27.7	$5.02\pm 0.38$	$0.40\pm 0.19$	$6.46\pm 0.49$
FB 2	0.075	35.5	$7.64\pm 0.59$	$0.37\pm 0.12$	$6.59\pm 0.51$
FB 3	0.05	48.3	$6.75\pm 0.52$	$0.36\pm 0.23$	$5.42\pm 0.42$

Abbreviations: $Y_{P/X}$ specific product titer, *STY* space-time yield

### Repetitive fed-batch

**Fig. 3:**
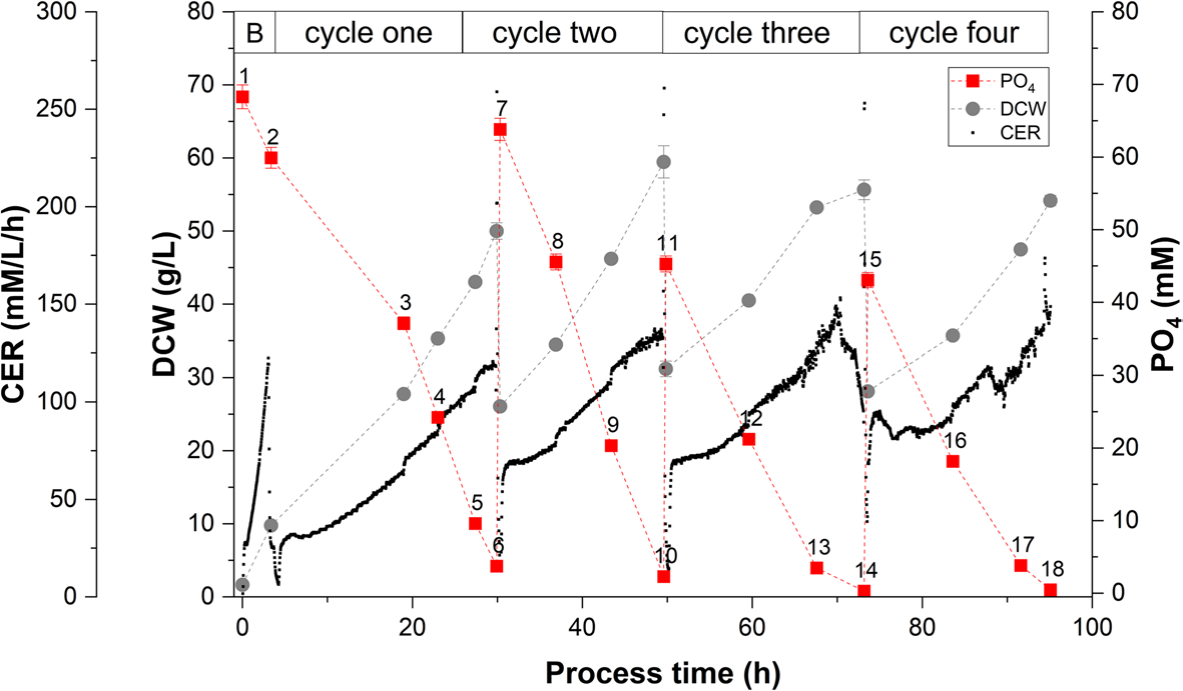
Repetitive fed-batch cultivation (RFB B): Four cycles (B=batch, cycles one, two, three with a partial harvest after each cycle, cycle four with full harvest; time series data of dry cell weight (DCW), extracellular PO_4_, carbon dioxide evolution rate (CER) over process time (h); PO_4_ in refill media was lowered after cycle two to adjust for 50 g/L DCW at harvest

The major challenge of the RFB was to derive a suitable cultivation strategy keeping the Fab productivity stable over several cycles. The extracellular PO_4_ concentration was defined as the harvest criterion according to the previous fed-batch characterization and was measured at-line. Two RFB strategies (referred to as RFB A and RFB B) were performed investigating whether a full PO_4_ limitation (below 1 mM) is feasible for repetitive cycles or not. RFB A approached the full PO_4_ limitation at harvest in cycle one, while RFB B approached the full PO_4_ limitation in the intermediate cycle three. Subsequently, the RFB B strategy is examined in detail, especially the cell physiology and productivity of the individual cycles, as the RFB A strategy indicated poor Fab productivity from cycle two. Four cycles were carried out in RFB B showing a high inter-cycle reproducibility of DCW and CER (Fig. 3). The first two cycles were harvested at the onset of PO_4_ limitation, while cycles three and four were harvested at full PO_4_ limitation (Table 2). The average length of each cycle was set to 22 h ($\pm 2 h$), only the first cycle took 30 h due to an initial biomass growth phase. Strain physiological parameters and Fab productivity were evaluated for each individual cycle (Table 2). The specific PO_4_ uptake rate $q_{PO4}$ was constant ($0.65\;mM/g_{DCW}/h$) over cycle one and two and decreased in the latter two cycles. During all cultivation cycles, the exponential feeding profile was set to $\mu _{set}=0.075\;1/h$ due to the highest achieved STY in FB 2 (Table 1). Nevertheless, the determined specific growth rate $\mu$ was falling below the setpoint, particularly in cycles three and four. Glucose accumulation was only noted at the end of cycle three resulting in temporary C-unlimited growth and higher $q_S$ in cycle four due to glucose carryover. C-balance calculations showed a progressive reduction in carbon recovery (cycle one: 0.93, cycle four: 0.75). Hereby, the $Y_{X/S}$ reduced significantly with successive cycles. The yield $Y_{CO2/S}$ remained constant throughout all cycles ($0.42\pm 0.03$ C-mol/C-mol). RFB B showed a stable product formation rate over three cycles with an average specific product formation rate between $q_{P,\,avg} = \left[ 0.2-0.3\right] \; mg/g_{DCW}/h$ yielding in a volumetric titer of 240 mg/L (Table 2). The amount of product harvested was calculated as an integral value over all cycles and was increasing up to a total of 615 mg in cycle four. Even though the Fab productivity was maintained over all four cycles, a decrease of the specific product formation rate $q_{P,\,avg}$ was observed with each additional cycle. Consequently, full PO_4_ limitation led to an increase of specific titer in cycle three ($4.4\;mg/g_{DCW}$) compared to the previous cycle on the cost of a significant drop of productivity in cycle four ($3.4\;mg/g_{DCW}$).

**Table 2:** RFB B comparison of individual repetitive fed-batch cycles with respect to PO_4_ concentration as harvest criterion (control parameter), strain physiological parameters and Fab productivity

	Cycle one	Cycle two	Cycle three	Cycle four
control parameter				
PO_4_$_{,\,end}$ (mM)	3.73	2.31	0.29	0.48
strain physiology				
${q_{PO4}}\;(mM/g_{DCW}/h)$	$0.065\pm 0.018$	$0.064\pm 0.015$	$0.035\pm 0.023$	$0.039\pm 0.023$
$\mu$ (1/h)	$0.076\pm 0.009$	$0.065\pm 0.007$	$0.048\pm 0.011$	$0.058\pm 0.018$
$q_{S}$ (C-mol/C-mol)	$0.154\pm 0.012$	$0.153\pm 0.013$	$0.139\pm 0.017$	$0.171\pm 0.022$
$Y_{X/S}$ (C-mol/C-mol)	$0.493\pm 0.048$	$0.425\pm 0.012$	$0.341\pm 0.053$	$0.333\pm 0.062$
$Y_{CO2/S}$ (C-mol/C-mol)	$0.413\pm 0.014$	$0.407\pm 0.026$	$0.425\pm 0.029$	$0.416\pm 0.066$
C-balance (-)	$0.93\pm 0.057$	$0.83\pm 0.018$	$0.77\pm 0.033$	$0.75\pm 0.01$
Fab productivity				
Product (mg/L)	$241.4\pm 18.1$	$241.3\pm 18.1$	$244.9\pm 18.4$	$183.8\pm 13.8$
$\sum$ Product harvested (mg)	$195.6\pm 14.7$	$337.9\pm 25.3$	$515.3\pm 38.6$	$614.5\pm 46.1$
$Y_{P/X}$ ($mg/g_{DCW}$)	$4.83\pm 0.38$	$4.06\pm 0.34$	$4.40\pm 0.35$	$3.39\pm 0.26$
$q_{P,\,avg}$ ($mg/g_{DCW}/h$)	$0.31\pm 0.065$	$0.21\pm 0.03$	$0.20\pm 0.069$	$0.15\pm 0.135$

Abbreviations: $PO_{4,\,end}$ PO_4_ concentration end of cycle (harvest), $q_{PO4}$ specific PO_4_ uptake rate, $q_S$ specific substrate uptake rate

**Fig. 4:**
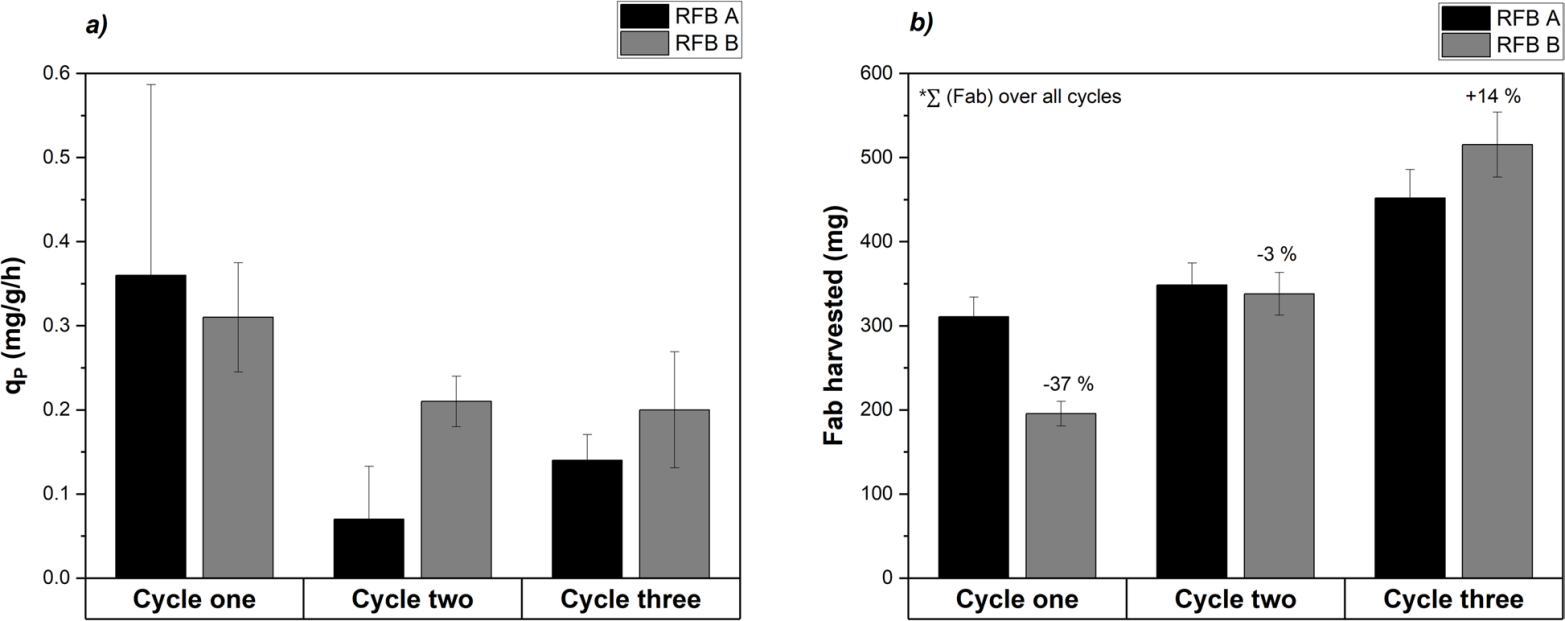
Comparison of two RFB cultivation strategies, RFB A (black) with full induction in cycle one, RFB B (gray) with full induction in cycle three: **a** average specific product formation rate $q_{P,\,avg}$ provided for each cycle comparing two different cultivation strategies, RFB A had high initial $q_{P,\,avg}$ with significant drop in cycle two and three, RFB B had lower initial $q_{P,\,avg}$ and higher cycle stability, **b** *Fab harvested was calculated as integral over cycles, RFB B cultivation led to 14% higher total Fab in cycle three compared to strategy RFB A under the tested conditions

In the next step, the cultivation strategy was altered as described (RFB A). Thereby, three cycles were carried out and compared to RFB B (cycle four not included here because of low productivity). Full PO_4_ limitation was introduced in the first cycle and the impact on productivity in the subsequent cycles was investigated (Fig. 4). Whereas, RFB A had a higher initial $q_{P,\,avg}$ of 0.36 $mg/g_{DCW}/h$ compared to RFB B as full PO_4_ limitation was approached in the first cycle, a significant drop of $q_{P,\,avg}$ was observed over the next cycles (Fig. 4a). Increased variations of $q_{P,\,avg}$ within cycle one and two are reflected in the larger standard deviations compared to RFB B. The total amount of Fab harvested was integrated over the cycles (Fig. 4b). Until cycle two RFB A was preferable due to a larger amount of the total Fab harvested (348 mg). However, RFB B led to an increase of harvested Fab by 14% after 3 cycles compared to RFB A ($515.3\pm 24.4$ vs. $451.8\pm 24.4$ mg, unpaired two-sided t-test, $p=0.0284$). Based on these observations, we derived a suitable cultivation strategy for the RFB: Targeting the onset of PO_4_ limitation until the last cycle maintained a stable volumetric titer, while full PO_4_ limitation in the final cycle provided a productivity boost (RFB B strategy). Thus, the PO_4_ level at harvest is a critical process parameter in RFB processing.

### Chemostat cultivation

**Fig. 5:**
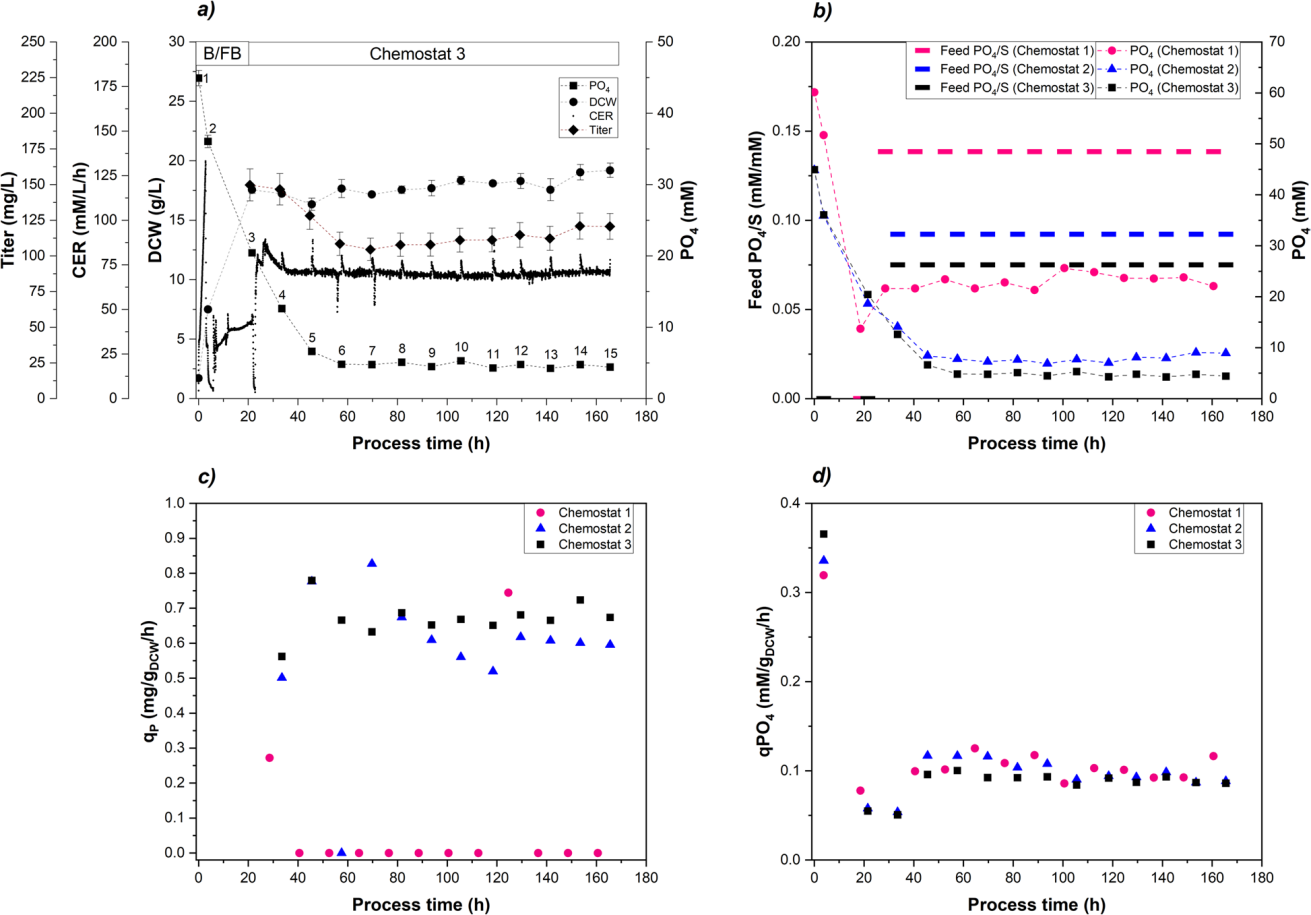
Chemostat cultivations (1, 2, 3) with targeted extracellular PO_4_ concentrations of 20 mM (pink), 10 mM (blue) and 5 mM (black): **a** Chemostat 3 cultivation details with extracellular PO_4_ (squares), DCW (circles), CER (dots), titer (diamonds); **b** PO_4_/S molar ratio in feed was control parameter (bars), extracellular PO_4_ was controlled parameter (dashed lines); **c** specific product formation rates **d** specific PO_4_ uptake rates

For initial chemostat cultivations the extracellular PO_4_ concentration during steady-state conditions was investigated by adjusting the PO_4_/S-ratio in the feed. $Y_{PO4/S}$ was determined to be 0.07 mM/mM (FB: Fig. 1, chemostat: Table 3). The substrate concentration in the feed was set to 50 g/L resulting in a biomass concentration of approximately 20 g/L assuming a theoretical biomass/substrate yield $Y_{X/S}$ of 0.4 g/g. Three cultivations were carried out (Chemostat 1, 2, 3) targeting 20, 10 and 5 mM extracellular PO_4_. The results of Chemostat 3, which had the highest productivity, are shown below (Fig. 5a). After a short transition phase following the switch from batch/FB to chemostat mode (samples 4-5), the cultivation parameters stabilized yielding in 18.2 g/L biomass concentration and 4.9 mM extracellular PO_4_. The CER remained constant throughout the chemostat cultivation. The volumetric titer was found to be at an average of $116\pm 12$ mg/L in chemostat operation, whereby a steady-state Fab productivity was achieved.

**Table 3:** Strain physiological parameters of chemostat cultivations 1-3: PO_4_/substrate ratio feed ($Y_{PO4/S; \;Feed}$) as control parameter, calculated PO_4_/S uptake ($Y_{PO4/S}$), extracellular PO_4_ supernatant (PO_4_) targeting 20, 10 and 5 mM (controlled parameter), specific PO_4_ uptake rate ($q_{PO4}$), C-balance

Cultivation	$\textbf{Y}_{\textbf{PO}_{\boldsymbol{4}}\mathbf {/S; Feed}}$ (mM/mM)	$\textbf{Y}_{\textbf{PO}_{\boldsymbol{4}}\mathbf {/S}}$ (mM/mM)	PO_4_ (mM)	$\textbf{q}_{\textbf{PO}_{\boldsymbol{4}}}$ (mM/g/h)	C-balance (-)
Chemostat 1	0.138	$0.066\pm 0.011$	$23.1\;(20^{\text {a}})\pm 1.4$	$0.104\pm 0.012$	$0.86\pm 0.05$
Chemostat 2	0.092	$0.063\pm 0.003$	$8.4\;(10^{\text {a}})\pm 1.9$	$0.097\pm 0.018$	$0.84\pm 0.05$
Chemostat 3	0.075	$0.057\pm 0.002$	$4.9\;(5^{\text {a}})\pm 0.7$	$0.088\pm 0.013$	$0.86\pm 0.03$

^a^ experimental value of extracellular PO_4_ (targeted PO_4_)

The PO_4_ concentration was controlled via continuous feeding at fixed PO_4_/substrate ratios in the range of 0.075 mM/mM (Chemostat 3) to 0.138 mM/mM (Chemostat 1) (Table 3). The washout of excessive PO_4_ occurred after two to three generation times (Chemostat 2, Chemostat 3) and steady-state conditions were reached (Fig. 5b). Chemostat 1 undershot the targeted value of 20 mM at the beginning, but a steady-state condition was reached within one generation time. The specific phosphate uptake rate $q_{PO4}$ was approximately 3.5 fold higher during batch cultivation and stabilized at 0.1 mM/g_DCW_/h during chemostat cultivation (Fig. 5d). Comparing the chemostat experiments, a slight decrease of $q_{PO4}$ was observed with a lowered PO_4_ concentration in the feed (Table 3). The growth rate was kept constant at 0.1 h^-1^ and the condition for a steady-state growth was fulfilled ($D=\mu$). As expected, a strong dependency of the specific product formation rates $q_P$ on the extracellular PO_4_ concentration was observed (Fig. 5c). Almost no productivity was measured in Chemostat 1 meaning that the promoter was repressed by an abundant PO_4_ availability ($q_P=0.08\;mg/g_{DCW}/h$). Chemostat 2 and 3 had a productivity of $q_P=\left[ 0.57-0.68 \; mg/g_{DCW}/h\right]$ induced by promoter de-repression under PO_4_ limitation. Chemostat 3 resulted in a more stable productivity, whereas Chemostat 2 showed fluctuations in the productivity, which can be seen in Fig. 5c and in the standard deviations of $q_P$ given in Table 4.

**Table 4:** Results of chemostat cultivations: manipulated factor was the extracellular PO_4_ concentration, the response variables were metrics describing Fab productivity

Cultivation	$\textbf{PO}_{\boldsymbol{4}\;\textbf{set}}$ (mM)	Y_P/X_ (mg/g)	q_P_ (mg/g/h)	STY (mg/L/h)
FB 2 (ref.)	$<1$	7.6	0.64^a^	6.6
Chemostat 1	20	$0.9\pm 1.9$	$0.08\pm 0.21$	1.7
Chemostat 2	10	$5.5\pm 2.0$	$0.57\pm 0.2$	7.4
Chemostat 3	5	$6.5\pm 0.8$	$0.68\pm 0.04$	8.6
Chemostat 4	1	$1.9\pm 2.9$	$0.08\pm 0.17$	2.2

^a^ reference FB 2 cultivation: $q_P$ is referring to Fab expression under PO_4_ limiting conditions

**Fig. 6:**
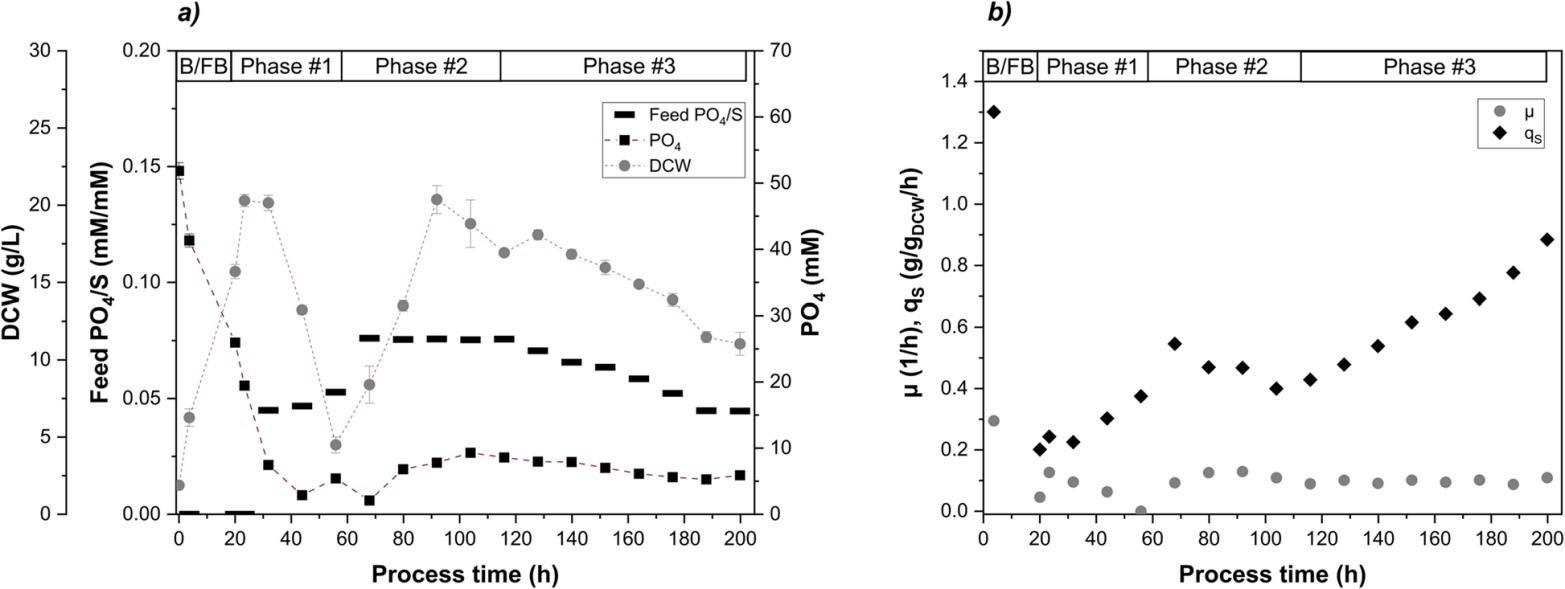
Chemostat cultivation 4: targeted PO_4_ concentration of 1mM with several phases, initial batch/fed-batch phase (B/FB) followed by 3 continuous phases, sharp reduction of PO_4_ in the feed (Phase #1), recovery phase with higher PO_4_/S feed supply (Phase #2), incremental reduction of PO_4_ in the feed (Phase #3); **a** control of PO_4_/S in the feed (black bars), extracellular PO_4_ (black squares), biomass concentration via DCW (gray circles); **b** specific growth rate $\mu$ (gray circles), specific substrate uptake rate $q_S$ (black diamonds)

As FB cultivations resulted in higher Fab productivity at full PO_4_ limitation, extracellular PO_4_ concentrations below 5 mM were investigated in the cultivation Chemostat 4. Two different strategies for targeting 1 mM PO_4_ were tested within Chemostat 4 in a dynamic mode (Fig. 6a). When the chemostat operation started, the feed was adjusted to the pre-calculated ratio of 0.05 mM/mM PO_4_/substrate at the given biomass concentration (Phase #1). Since cell washout and substrate accumulation were observed as a consequence of Phase #1, the feed ratio was adjusted to 0.075 mM/mM ensuring a recovery and stabilization of the cultivation (Phase #2). Biomass recovered during Phase #2 back to the initial value of 20 g/L and extracellular PO_4_ stabilized at 7 mM. Finally, an incremental reduction of PO_4_ by $0.5\%\;1/h$ in the feed was implemented (Phase #3). The incremental reduction allowed to study the cultivation at an quasi-equilibrium stage with only minor changes over time. Interestingly, no complete cell washout was observed in Phase #3. The system responded to phosphate limitation with a reduction in biomass concentration (20 g/L to 11 g/L). Since no substrate accumulation was measured during this phase, the remaining cells thus had more phosphate available at a simultaneously increased substrate uptake rate $q_S$ (Fig. 6b) and at a decreased $Y_{X/S}$ (data not shown). The Fab productivity was significantly lower compared to Chemostat 3 and this cultivation strategy was not feasible (Table 4).

The two-stage cascaded cultivation had a negligible productivity in the first stage, as it was intended to produce biomass for stage two. The product-producing reactor resulted resulted in a fluctuating productivity between 0 and 0.6 $mg/g_{DCW}/h$. The overall STY of this strategy was 2.3 mg/L/h (supporting information C).

## Discussion

Intensified cultivations with induced *E. coli* systems are often instable in their target molecule production due to non-producing subpopulations arising over process time [21]. Most studies dealing with auto-inducible promoters for recombinant Fab production focus on strain engineering, media development and process parameter screening in shake flask or FB cultivations [26, 47, 48]. Further process development of Fab production with *E. coli* including strategies for process intensification is still poorly covered and only a few examples were reported, non of them using an auto-inducible expression system [11, 19].

This study investigated the application of the PO_4_-sensitive, auto-inducible expression system for recombinant Fab production using *E. coli* W3110 following a two stage approach: First, a physiological characterization obtained from FB cultivations revealed that PO_4_ limitation (PO_4_<6.1mM) caused a decrease of the cell-specific growth rate and substrate uptake, elevated CO_2_/substrate yield and an increased Fab productivity. After all extracellular PO_4_ was depleted, results revealed a complete breakdown of the cellular metabolism presumably due to a cellular PO_4_ limitation, which is consistent with literature [28, 42]. Second, intensified processing strategies (RFB, chemostat) were investigated based on the obtained strain physiological parameters aiming to improve the cultivation space-time yield. For industrial application relevant factors were assessed: stability of Fab productivity over time, cell washout in continuous cultivations, substrate accumulation and by-product formation.

We found that three distinct phases of PO_4_ availability can be distinguished during FB cultivation: i) PO_4_ excess above 6.1 mM, ii) onset of PO_4_ limitation between 6.1 and 3.4 mM and iii) full PO_4_ limitation below 1.0 mM (Fig. 1). In detail, the observed downward shift in $Y_{PO4/S}$ under decreasing extracellular PO_4_ concentrations (Fig. S3) was attributed to the intracellular PO_4_ storage, which allowed a partial decoupling of PO_4_ uptake and substrate uptake. Notably, the decrease of $Y_{PO4/S}$ preceded other detectable changes in metabolism and growth rate, suggesting that $Y_{PO4/S}$ represents a sensitive physiological marker for the onset of PO_4_ limitation (Fig. 1 and Table 3). Cellular PO_4_ is crucial for sustaining metabolic functions including ATP generation and nucleic acid synthesis [49, 50]. We assumed, that the intracellular PO_4_ storage temporarily sustained metabolic demands. Intracellular PO_4_ measurements in this study revealed 3.0 wt.% PO_4_/DCW under PO_4_ excess, decreasing to 0.3 wt.% under full PO_4_ limitation (Table S3). This finding is in close agreement with a previous elemental composition analysis of an *E. coli* W strain, which reported 2.9 wt.% PO_4_ (under extracellular PO_4_ excess) [51]. Even though *E. coli* mediates the limitation below PO_4_ concentrations of $4\, \mu M$ through activation of the Pho regulon [23, 25], we observed a cellular response already at 6.1 mM extracellular PO_4_ independent from the cultivation mode, which has not been shown in literature yet to our knowledge (Fig. 1 and Table 3). Schuhmacher et al. reported that the ATP synthase activity declined below 4.2 mM PO_4_ leading to reduced cell growth, but elevated substrate uptake and respiratory activity in FB cultivations [42]. Other studies support these findings and showed that PO_4_ limitation reduced cellular ATP availability, leading to decreased biomass formation despite increased substrate uptake and respiratory activity. Marzan et al. demonstrated that PO_4_ limitation induced a metabolic shift by PhoB- and ArcAB-dependent regulation, which repressed TCA cycle activity and favored maintenance metabolism over cellular growth [52]. Likewise, Pandi et al. demonstrated that PO_4_ limitation triggered a regulatory response involving the PhoB/PhoR system and global carbon regulators, resulting in downregulation of biosynthetic and other energy-intensive pathways [53]. Considering the FB 1 cultivation presented (Strain physiological characterization in fed-batch operation section), a significant reduction of cell growth was observed under full PO_4_ limitation, while the specific substrate uptake $q_S$ was stable upon the metabolic breakdown (Figs. 1b, S2). At the translational level it was reported, that PO_4_ limitation in chemostat cultivations constrained ribosome biogenesis, as PO_4_-limited *E. coli* cells exhibited approximately two-fold lower ribosome-to-protein (R/P) ratios compared to C-limited cells at comparable growth rates [54]. The reduction in ribosomes was attributed to a limited nucleotide availability under PO_4_ limitation. Still literature states, that the cellular protein production did not seem to be impacted by fewer ribosomes, indicating that the ribosomes were utilized more efficiently under PO_4_ limitation. The high carbon recovery ($>0.9$) of samples 1-4 during FB cultivation indicated a proper functionality of the cellular metabolism (Fig. S1). Although, overall acetate formation as a major by-product of overflow metabolism was very low, acetate accumulation was observed at the end of the cultivation at $Y_{Ac/S}=0.04$ C-mol/C-mol (sample 7). With the onset of PO_4_ limitation (sample 5-7), a further decreased $Y_{X/S}$ yield was observed, while full PO_4_ limitation (sample 6-7) led to an increased $Y_{CO2/S}$ yield. Since less biomass was generated, excess carbon might be reallocated towards cellular maintenance pathways [55]. The same trend was observed for RFB B, where $Y_{X/S}$ and the total carbon recovery decreased with each additional cycle indicating an inefficient carbon utilization related to PO_4_ limitation in cycle three and four (Table 2). Even though the exponential feeding profile was kept constant over all cycles, $\mu$ and $q_S$ were below the setpoint especially during cycle three. In combination with the calculated C-balance and the underlying yields $Y_{X/S}$ and $Y_{CO2/S}$ (where $Y_{Ac/S}$ negligible), it is evident that the cell growth was negatively impacted with each additional cycle conducted. Nevertheless, RFB processing also revealed a quick recovery of cell growth ($\mu$) and respiratory activity (data not shown) upon addition of PO_4_ to fully PO_4_ limited cells (Fig. 3), which is in alignment with literature [25]. The cycle length of the RFB cultivations was a controlled parameter and predetermined by the biomass and PO_4_ concentration at the beginning of each cycle as well as the specific feeding rate $\mu$ (kept constant at $\mu _{set}=0.075\;1/h$). The lower the initial biomass and the higher the initial PO_4_ concentration, the longer a cycle took to fulfill the harvest criteria. However, cycle length ($22\pm 2\;h$) and final biomass concentration ($55\pm 4\;g/L$) were not feasible as harvest criteria due to variations exceeding the narrow limit of harvest (Fig. 3). Instead, at-line monitoring of the extracellular PO_4_ concentration emerged as the only reliable strategy for defining harvest points in the RFB cultivation. The time delay (30 min) associated with the colorimetric PO_4_ quantification (Cedex Bio HT Analyzer) limits the applicability of robust closed-loop process control. While Karnachoriti et al. proposed Raman spectroscopy as a promising tool for real-time nutrient monitoring in bioreactors, the detection limit of PO_4_ reported in this study was not sufficient for our control strategy [56]. Alternatively, the implementation of a state observer approach for estimating PO_4_ based on available on-line signals (e.g., CER, OUR, NH_3_) represents a promising route for monitoring and control [57, 58]. While FB and RFB cultivations provided insights into the dynamic cellular responses to PO_4_ limitation (e.g., $Y_{PO4/S}$ and $q_P$), subsequent chemostat experiments were conducted to examine these responses under steady-state cultivation conditions. Different PO_4_/S ratios of the feed were tested in four chemostat experiments (Chemostat 1 - 4). Hereby, the dilution rate was kept constant at a setpoint of $D=0.1\;1/h$. Cell washout was only observed when the feed was abruptly switched to the lowest (PO_4_/S ratio of 0.05 mM/mM (Fig. 6, Phase #1). In contrast, when the PO_4_/S ratio was gradually decreased, allowing the system to reach a quasi-equilibrium, the biomass concentration declined by 50% (Phase #3). This suggests that the biomass reduction was an adaptive response to PO_4_ limitation. With decreasing total biomass, more PO_4_ became available per cell, potentially allowing the remaining population to keep up cell division rates [59]. Since the maximum specific substrate uptake rate of $q_{S,\,max}=1.2\,g/g_{DCW}/h$ was not exceeded, no substrate accumulation was detected in the supernatant during Phase #3 [58, 60]. Chemostat cultivations 1, 2, and 3 were operated at various PO_4_ levels and demonstrated stable growth (Fig. 5). However, when passing below the extracellular PO_4_ limit of 5 mM, no stable long-term cultivation could be established (Chemostat 4). We attributed this to a functional threshold required for cellular growth.

Interestingly, product formation was favored under conditions of reduced metabolic load. FB experiments showed that low cell-specific growth rates $\mu = \left[ 0.05-0.1\right] \; 1/h$ promote soluble Fab expression, aligning with previous observations in Fab-producing *E. coli* strains [61, 62]. These conditions could have mitigated the burden on periplasmic protein expression and folding compared to cytoplasmic expression [63]. A previous study reported that the periplasmic product retention of a Fab was improved by lowering the cell-specific feed rate, since the outer membrane leakage of the Fab to the medium was reduced [29]. The expression system based on the phoA promoter was induced under PO_4_-limited conditions, which is reflected in the increased $q_P$ levels (Fig. 1b). This interpretation is further supported by RT-qPCR data showing an upregulation of *phoB* and the gene of interest under PO_4_ limitation, which confirms the transcriptional activation of the phoA promoter (Fig. 2). The subsequent decline in transcript levels under prolonged, full PO_4_ limitation is consistent with reduced metabolic activity and decreasing productivity (Fig. 1). Furthermore, Fab expression was already noticed above 20 mM PO_4_ during FB and RFB cultivations, a phenomenon referred to here as leaky expression (Figs. 1a, 3) and described in literature [13, 64]. Under leaky expression conditions, $q_P$ reached $0.3\; mg/g_{DCW}/h$ in both FB and RFB modes and $q_P$ doubled under full PO_4_ limitation, which is consistent with a previous study [28]. From a process engineering perspective, moderate product formation under PO_4_ excess conditions may be advantageous to balance protein expression and segregation/folding in the periplasm [65]. At the same time, this leaky expression indicated that the phoA promoter was not fully repressed under PO_4_ excess conditions, which reduces strict on–off controllability but also lowers abrupt induction stress compared to tightly regulated systems. Reported titers for recombinant Fab production vary widely in the range from a few mg/L to 2 g/L, reflecting the strong influence of host strain, expression system, produced Fab of interest and process conditions [10, 13]. Fink et al. screened 32 *E. coli* production clones for periplasmic Fab expression in FB cultivations and obtained specific titers up to $7.4\;mg/g_{DCW}$ using the strains HMS174(DE3) and BL21(DE3) with a T7-based expression system [11]. We determined specific Fab titers in the range of $5.0-7.6\;mg/g_{DCW}$ depending on the feed rate in FB mode (Table 1). In RFB cultivations, the $q_{P,\,avg}=0.3\; mg/g_{DCW}/h$ in the first cycle reflected leaky expression under PO_4_ excess conditions as full limitation was intentionally avoided (Table 2). The subsequent decline in $q_{P,\,avg}$ during cycles two and three (and four, RFB B only) is likely due to the accumulation of non-producing subpopulations, a known challenge in prolonged cultivations depending on the metabolic load [38, 66]. The cells underwent 18 generations including pre-culture and RFB cultivation with a high metabolic load under PO_4_ limitation. Insufficient biomass renewal after each cycle may have contributed to this effect, as previously induced cells were partially carried over into subsequent cycles. Hereby, adjusting the ratio of remaining cells and fresh media added to the reactor might mitigate the carry over effect. We further hypothesized that full PO_4_ limitation in cycle three led to a breakdown of the expression machinery, explaining the sharp productivity drop in cycle four. In addition, the initial PO_4_ supplementation at the beginning of each cycle may have repressed the promoter due to excess PO_4_ availability. These findings emphasize the need to balance induction strength with expression stability, suggesting that PO_4_ limitation should not fall below 1 mM during RFB processing (excluding the last cycle) [21]. Chemostat cultivations indicated that Fab productivity was tightly linked to the PO_4_/S ratio of the feed adjusting for the extracellular PO_4_ concentration respectively (Fig. 5). While 20 mM PO_4_ repressed Fab expression, de-repression occurred at 10 – 5 mM already with a stable productivity up to $0.68 \; mg/g_{DCW}/h$ (Table 4). By regulating the induction strength via the supplied PO_4_/S feed ratio, the productivity was maintained over 34 generations. Nevertheless, mutational escape represents a limitation of long-term microbial cultivations under strong metabolic burden [67]. In this study, Chemostat 4 exhibited a stable productivity $q_P$, indicating that no productivity-hampering mutations under the applied conditions occurred. It should be noted that industrial-scale biomanufacturing processes may encompass 100 or even more generations due to the necessary scale-up steps. This exceeds the time horizon investigated here and increases the likelihood of genetic instability [21]. However, several aspects of the proposed processing strategy may contribute to mitigating selective pressure for non-producing mutants. First, the expression was based on an auto-inducible promoter with tunable, moderate expression levels, as previously reported for auto-inducible systems in literature [68]. Second, cultivating at a reduced temperature (30°C) is a commonly applied strategy to reduce the metabolic burden [65]. Nevertheless, mutational escape cannot be fully excluded, particularly over extended operation times. Schuller et al. previously investigated the long-term stability of Fab production in chemostat cultures (21 generations) with two different *E. coli* strains and reported specific titers between $1-4\;mg/g_{DCW}$ [40]. In our chemostat experiments, specific Fab titers of up to $6.5\;mg/g_{DCW}$ were achieved, corresponding to 86% of the maximum specific titer obtained in FB processing. Moreover, chemostat operation increased the space–time yield by 30% compared to the best-performing FB 2 ($8.6\pm 0.41$ vs. $6.6\pm 0.31$ mg/L/h, unpaired two-sided t-test, $p=0.0025$). RFB B resulted in a STY of 6.3 mg/L/h after three cycles, while FB2 reached a higher STY of 6.6 mg/L/h. Under the assumption of an ideal RFB cultivation by using the average productivity achieved throughout cycle one as a hypothetical constant $q_{P,\,avg}$ value, a space-time yield of 7.7 mg/L/h would be attainable, which represents a promising alternative to the conventional FB process as well [35]. Overall, the chemostat cultivation achieved the highest STY among all tested process strategies.

The findings of this study represent a good starting point for future process parameter exploration and optimization to further improve the STY. Cultivation strategies for the auto-inducible phoA system under PO_4_ limitation were assessed by varying single factors across different processing modes. Notably, the RFB strategy represents a promising concept in comparison to the well-established FB mode. Successive cultivation cycles were operated within a defined physiological window, thereby temporally decoupling biomass generation and auto-induction while minimizing CIP/SIP-related downtime. Building on these results, we further aim to implement a model-based process monitoring strategy for the on-line determination of PO_4_ allowing ideal harvest time points in RFB operations and reducing manual sampling efforts. Rather than relying on time-delayed off-line analytics, the integration of a bioprocess model with a soft-sensor algorithm utilizing measurements of proxy variables could offer robust real-time process control. Future design of experiment (DoE) approaches could elucidate process parameter interactions and test process robustness (e.g., cell washout characteristics in chemostat) [69].

## Conclusions

This study explored intensified processing strategies of the pharmaceutical relevant Fab Ranibizumab using an auto-inducible *E. coli* W3110 strain. While PO_4_ limitation triggered product formation, it also strongly impacted cell growth, carbon utilization and substrate uptake. The dual role of PO_4_ as an inducer and critical nutrient was considered for implementing intensified processing strategies. This work demonstrated that PO_4_ availability can be used as a physiological control variable to regulate auto-induction, productivity, and process stability across multiple cultivation modes. An important contribution of this study was the identification and experimental validation of a functional PO_4_ threshold that enables reliable phoA-mediated induction while maintaining stable cellular physiology. This threshold-based control principle proved to be transferable from fed-batch to repetitive fed-batch and chemostat operation, despite their fundamentally different dynamic and steady-state characteristics. Continuous processing was found to improve the space-time yield by +30% compared to conventional fed-processing, allowing potentially more economic Fab production in the future.

## Supplementary Information

Supplementary Material 1.

## Data Availability

The datasets generated and/or analysed during the current study are available from the corresponding author on reasonable request.
