# Supplementary material for: Intensified upstream processing by a phosphate-regulated, auto-inducible expression system in E. coli W3110 for recombinant Fab production
Source: BMC Microbiol. 2026 Mar 11;26:375. doi: 10.1186/s12866-026-04887-y (PMC13088625; doi:10.1186/s12866-026-04887-y)
Supplement: Supplementary file 1 — Supplementary Material 1. [file 12866_2026_4887_MOESM1_ESM.pdf]

Supporting Information

Intensified upstream processing by a phosphate-regulated, auto-inducible expression system in *E. coli* W3110 for recombinant Fab production

Rüdiger Lück[1], Oliver Spadiut[1], Julian Kopp[1,*]

[1]Integrated Bioprocess Development, TU Wien, Getreidemarkt 9, 1060 Vienna, Austria
[*]Correspondence: julian.kopp@tuwien.ac.at (orcid.org/0000-0003-4975-7103)

A Feed and media

The minimal cultivation media was prepared according to *DeLisa* [44]. Hereby, the PO_4 concentration in the media and feed was adapted towards the required extracellular PO_4 concentrations (Tab. S1). Chemostat 4 utilized two separate feed lines that enabled a flexible adaption of the PO_4/S ratio in the feed. One feed had a PO_4/S ratio of 0.075 mM/mM corresponding to 5 mM extracellular PO_4, the second feed contained no PO_4. Trace elements and the selection marker were prepared separately for each media (Tab. S2). FB and RFB cultivations were carried out using an exponential feed profile according to Eq. 1.

$$F(t) = \frac{\mu \cdot \rho_{Feed} \cdot X(t)}{Y_{X/S} \cdot c_{Feed}} \quad (1)$$

F(t) feed rate (g/h); μ specific biomass growth rate (1/h); ρ_{Feed} feed density (g/L); X(t) biomass (g); $Y_{X/S}$ biomass/substrate yield; c_{Feed} feed concentration (g/L)

Table S1: Media and feed composition of fed-batch, RFB and chemostat cultivations

**Media / Feed** (g/L)	**FB**	**RFB**	**Chemostat 1**	**Chemostat 2**	**Chemostat 3**	**Chemostat 4**
$C_6H_{12}O_6 \cdot H_2O$	22 (B)<br>440 (FB)	440	55	55	55	55
KH_2PO_4	2.1 (B)	3.8	1.5	0.9	0.7	0 | 0.7 (*)
$(NH_4)_2HPO_4$	6.3 (B)	11.5	4.4	2.8	2.3	0 | 2.3 (*)
$C_6H_8O_7$	1.7 (B)	1.7	1.7	1.7	1.7	1.7

(*): two parallel, separate feeds were used for Chemostat 4

Table S2: Trace elements and selection marker prepared separately

**Trace element**	**final concentration** (g/L)
$MgSO_4 \cdot 7\,H_2O$	1.2000
Fe(III)citrate	0.1000
EDTA	0.0084
$Zn(CH_3COO)_2 \cdot 2\,H_2O$	0.0130
$CoCl_2 \cdot 6\,H_2O$	0.0025
$MnCl_2 \cdot 4\,H_2O$	0.0150
$CuCl_2 \cdot 2\,H_2O$	0.0012
H_3BO_3	0.0030
$Na_2MoO_4 \cdot 2\,H_2O$	0.0025
Thiamine HCl	0.0045
Tetracycline HCl	0.0100

B Fed-batch

The carbon-balance was calculated between discrete sampling times including the biomass/substrate yield $Y_{X/S}$ (orange)), carbon dioxide/substrate yield $Y_{CO_2/S}$ (green)) and acetate/substrate yield $Y_{Ac/S}$ (violet) (Fig. S1).

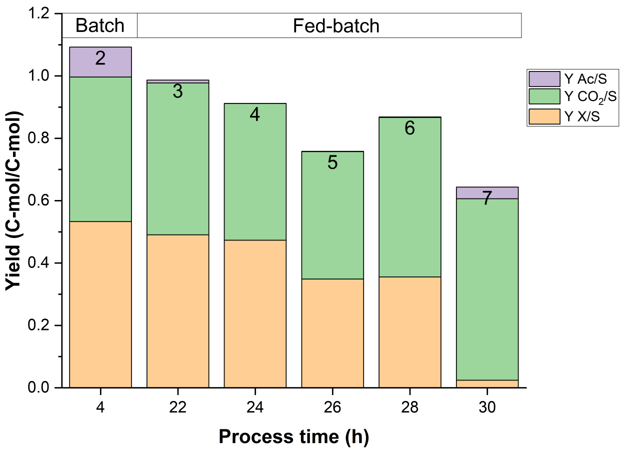

Figure S1: Fed-batch cultivation: carbon-recovery over process time including biomass/substrate yield $Y_{X/S}$, carbon dioxide/substrate yield $Y_{CO_2/S}$ and acetate/substrate yield $Y_{Ac/S}$

Glucose accumulation was only observed under full PO_4 limitation below 1.0 mM. The cultivation FB 1 showed a significant glucose accumulation, as it was prolonged even when the CER signal already indicated a breakdown (sample 6) of cellular metabolism to capture the response (Fig. S2).

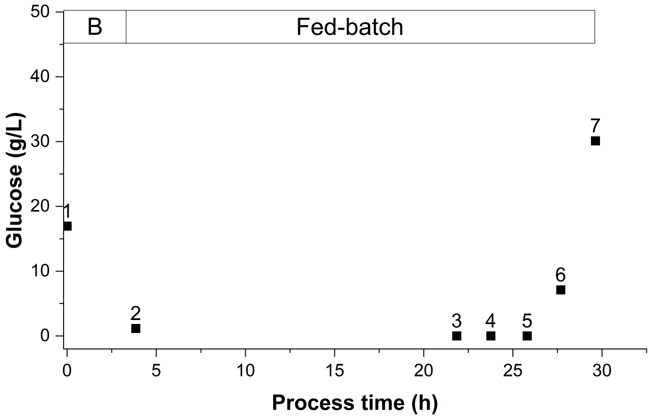

Figure S2: FB 1 cultivation: time series of glucose concentration over process time

The uptake ratio $Y_{PO4/S}$ as a physiological marker for PO_4 limitation was stable during PO_4 excess at $Y_{PO4/S} = 0.07\, mM/mM$. During the onset of PO_4 limitation, $Y_{PO4/S}$ started to decrease (3.4-6-1 mM extracellular PO_4). The product formation rate qP increased 2-fold under full PO_4 limitation (below 1 mM extracellular PO_4). Data from all fed-batch cultivations are summarized in the plot below (Fig. S3).

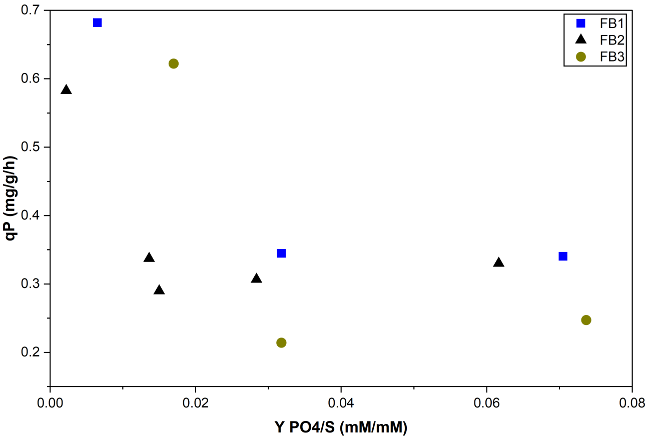

Figure S3: Comparison of fed-batch cultivations FB1, FB2 and FB3 with respect to PO_4/S uptake ($Y_{PO4/S}$) and product formation rate (q_P); beginning of PO_4 limitation indicated by decrease of $Y_{PO4/S}$ between 3.4 - 6.1 mM PO_4; elevated product formation rates q_P were observed below 1 mM PO_4

Extracellular and intracellular PO_4 concentrations were determined at-line using colorimetric assay kits on a Cedex Bio HT Analyzer (Roche, Basel, Switzerland). For intracellular measurements, cells were lysed by high-pressure homogenization (HPH) in a PO_4-free buffer (100 mM Tris-HCl, 5 mM EDTA, 5 mM benzamidine-HCl monohydrate, pH 7.2). To prevent carry-over of extracellular PO_4, the cell pellet was washed with the same buffer, centrifuged (14,000 rpm, 20 min, 4 °C), and re-suspended prior to HPH. The resulting intracellular PO_4 concentration was normalized to the dry cell weight (DCW) to calculate the mass fraction PO_4/DCW (Tab. S3). A composition analysis of an *E. coli* W strain determined 2.9 wt.% of phosphorous, which aligned with FB sample 3 (*) under PO_4 excess [51].

Table S3: Extracellular and intracellular PO_4 concentrations determined in fed-batch cultivations

**Sample**	**DCW** (g/L)	**PO_4 extracellular** (mM)	**PO_4 intracellular** (mM)	**spec. intracellular PO_4** (mg/g_{DCW}) | (wt.%)
FB sample 3 (*)	21.6	44.6	6.9	30.3 | 3.0 %
FB 1 sample 3	41.7	15.3	7.2	16.4 | 1.6 %
FB 1 sample 5	52.4	1.0	2.6	4.6 | 0.5 %
FB 1 sample 7	50.1	0.2	1.6	3.1 | 0.3 %

(*): additional fed-batch cultivation not shown in this study

C Cascaded processing

The cascaded cultivation, which spatially separated biomass growth and product formation resulted in a lower cell-specific productivity qP and STY compared to the other processing modes evaluated (Fig. S4). Although steady-state conditions were achieved with respect to the targeted extracellular PO_4 concentrations, the productivity of the second stage fluctuated between $0-0.6$ $mg/g_{DCW}/h$. We attribute these fluctuations to the intracellular PO_4 storage effects, which may delay or destabilize the activation of the phoA promoter as cells transition from conditions

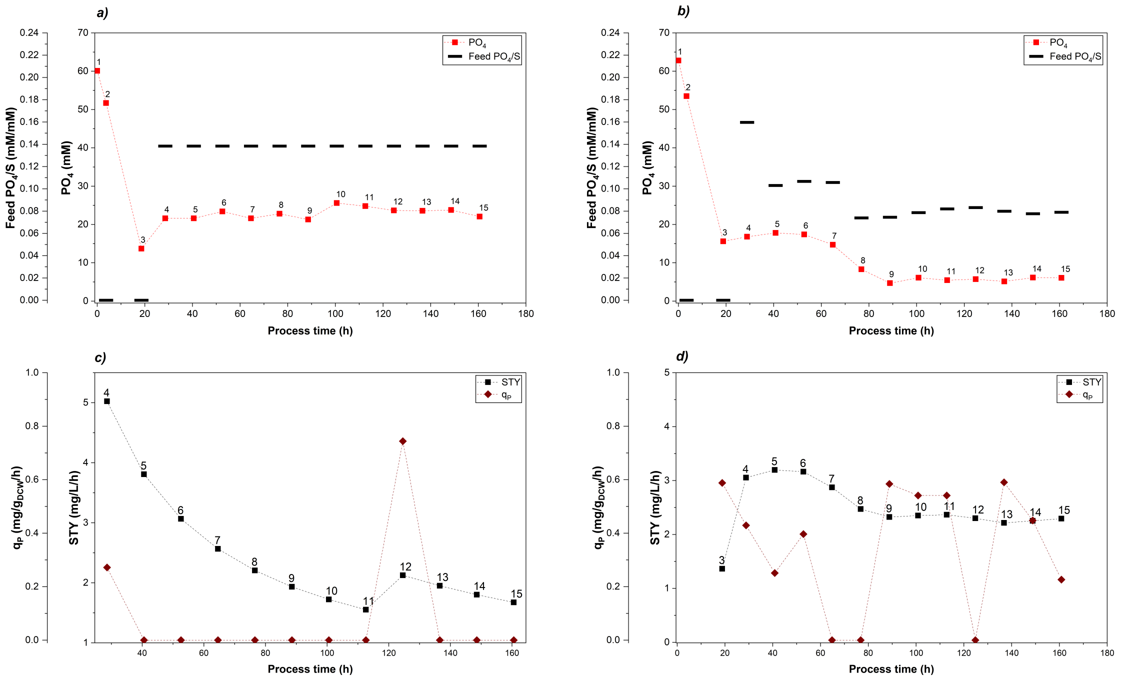

Figure S4: Cascaded processing strategy with two-stage bioreactor setup. a) extracellular PO_4 was adjusted in the first stage for 20 mM by setting the PO_4/S feed to 0.14 mM/mM, b) adjusting the feed of the second stage for targeting different extracellular PO_4 concentrations, c) cell-specific productivity q_P and STY of first stage, d) cell-specific productivity q_P and STY of second stage

of PO_4 excess in the first stage to PO_4 limited conditions in the second stage. Such intracellular buffering of PO_4 could result in transient promoter de-repression and thereby prevent stable Fab production in the cascaded setup.
